# Antidepressant-like Effect of Tetrahydroisoquinoline Amines in the Animal Model of Depressive Disorder Induced by Repeated Administration of a Low Dose of Reserpine: Behavioral and Neurochemical Studies in the Rat

**DOI:** 10.1007/s12640-013-9454-8

**Published:** 2014-01-10

**Authors:** Lucyna Antkiewicz-Michaluk, Agnieszka Wąsik, Edyta Możdżeń, Irena Romańska, Jerzy Michaluk

**Affiliations:** Department of Neurochemistry, Institute of Pharmacology Polish Academy of Sciences, 31-343 Kraków, Poland

**Keywords:** Depression-reserpine model, Forced swim test, Tetrahydroisoquinolines, Monoamines metabolism, Brain structures, Rat

## Abstract

Animal models are widely used to study antidepressant-like effect in rodents. However, it should be mentioned that pharmacological models do not always take into account the complexity of the disease process. In the present paper, we demonstrated that repeated but not acute treatment with a low dose of reserpine (0.2 mg/kg i.p.) led to a pharmacological model of depression which was based on its inhibitory effect on the vesicular monoamine transporter 2, and monoamines depleting action in the brain. In fact, we observed that chronic treatment with a low dose of reserpine induced a distinct depressive-like behavior in the forced swim test (FST), and additionally, it produced a significant decrease in the level of dopamine, noradrenaline, and serotonin in the brain structures. 1,2,3,4-Tetrahydroisoquinoline (TIQ) and its close methyl derivative, 1-methyl-1,2,3,4-tetrahydroisoquinoline (1MeTIQ) are exo/endogenous amines present naturally in the mammalian brain which demonstrated a significant antidepressant-like effect in the FST and the reserpine model of depression in the rat. Both compounds, TIQ and 1MeTIQ, administered chronically in a dose of 25 mg/kg (i.p.) together with reserpine completely antagonized reserpine-produced depression as assessed by the immobility time and swimming time. Biochemical data were in agreement with behavioral experiments and demonstrated that chronic treatment with a low dose of reserpine in contrast to acute administration produced a significant depression of monoamines in the brain structures and impaired their metabolism. These neurochemical effects obtained after repeated reserpine (0.2 mg/kg i.p.) in the brain structures were completely antagonized by joint TIQ or 1MeTIQ (25 mg/kg i.p.) administration with chronic reserpine. A possible molecular mechanism of action of TIQ and 1MeTIQ responsible for their antidepressant action is discussed. On the basis of the presented behavioral and biochemical studies, we suggest that both compounds may be effective for the therapy of depression in clinic as new antidepressants which, when administered peripherally easily penetrate the blood–brain barrier, and as endogenous compounds may not have adverse side effects.

## Introduction

In recent years, depression has been recognized as a major public health problem. Understanding how to prevent and treat depression is therefore, an urgent subject. It is well known that monoamine neurotransmitters, such as dopamine (DA), noradrenaline (NA), and serotonin (5-HT) in the central nervous system play a key role in the pathophysiology of depression (Cantello et al. 1989 Chan-Palay and Asan 1989; Colpaert 1987; Mayeux et al. 1984; Elhwuegi 2004). However, abnormalities in monoaminergic neurotransmission are associated with a number of neurological disorders including Parkinson’s disease (PD) and schizophrenia. Although the mechanism provoking depression has not been clearly elucidated; however, oxidative stress associated with generation of reactive oxygen species (ROS) can be one of the main causes in molecular processes underlying this disease. The endogenous generation of ROS results from metabolism of monoamines in the cytosol and auto-oxidation of monoamines. Physiologically, neurons have many endogenous mechanisms to maintain health and protect against degeneration. The vesicular monoamine transporter 2 (VMAT2) is one of such custodians that function to regulate the cytosolic environment of neurons, protecting them from endogenous and exogenous toxins (Uhl 1998; Miller et al. 1999). Localized on vesicular membranes in neurons, VMAT2 acts to accumulate cytosolic monoamines in synaptic vesicles after they have been synthesized from their precursors for regulated exocytotic release as well as after their re-uptake from the synaptic cleft into the neuron (Surratt et al. 1993). The monoamines, particularly DA and NA have the ability to undergo spontaneous oxidation in the cytosol, which is potentially damaging to cellular structures (Graham 1978; Antkiewicz-Michaluk et al. 2006; Wąsik et al. 2009). Thus, the level of VMAT2 expression plays an important role in nerve cell safety and is essential for cellular susceptibility to oxidation (Liu et al. 1992). In fact, in VMAT2-deficient mice the striatal DA level was reduced by 85 % with a concomitant reduction in the metabolites, DOPAC and HVA. In addition, several markers of oxidative stress and damage were observed in the VMAT2-deficient mice. Moreover, it was found that disruption of VMAT2 led to depressive-like phenotypes (Ziemssen and Reichmann 2007; Taylor et al. 2009).

**Fig. 1:**
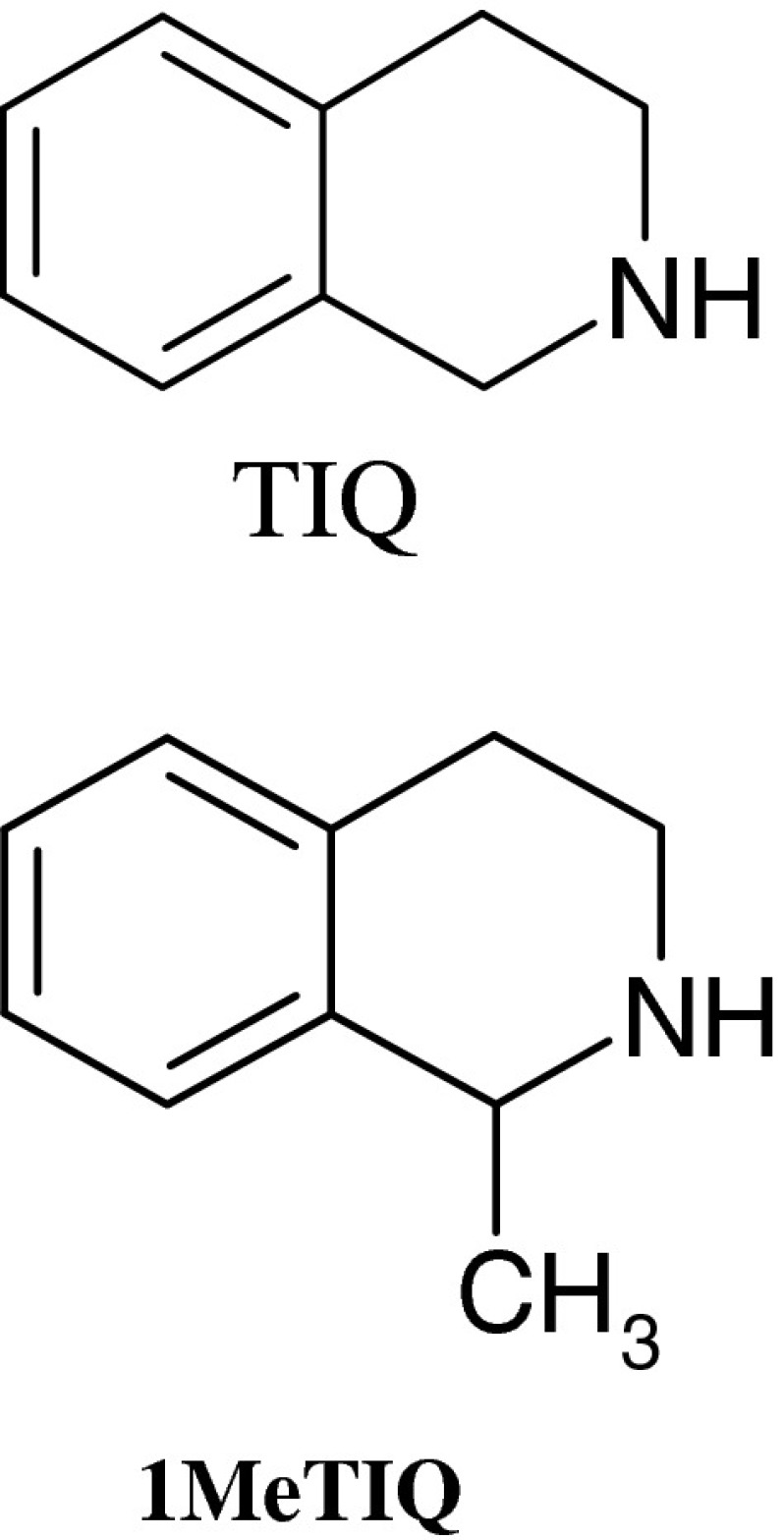
Chemical structure of TIQ and its close derivative, 1MeTIQ

Reserpine is a vesicular monoamine re-uptake blocker, which depletes monoamines in the brain, and produces depression-like syndrome in animals (Kandel 2000; Nagakura et al. 2009; Rojas-Corrales et al. 2004). Thus, it seems to be an ideal model to screen the potential antidepressants. In the present study, we analyzed the antidepressant potential of endogenous substances from the tetrahydroisoquinoline group: 1,2,3,4-tetrahydroisoquinoline (TIQ) and its close methyl-derivative, 1-methyl-1,2,3,4-tetrahydroisoquinoline (1MeTIQ; Fig. 1). Both these compounds are members of the TIQ family widespread in plant, animal, and human brains (McNaught et al. 1998; Rommelspacher and Susilo 1985). Among several endogenous TIQs, 1MeTIQ has a special position as a neuroprotective compound with antiparkinsonian potential, since it was demonstrated to reverse bradykinesia induced by 1-methyl-4-phenyl-1,2,3,6-tetrahydropyridine (MPTP) or 1-benzyl-1,2,3,4-tetrahydroisoquinoline (1BnTIQ) (Makino et al. 1990; Kotake et al. 1995; Tasaki et al. 1991). Both TIQ and 1MeTIQ, in contrast to other TIQs (e.g., 1BnTIQ and salsolinol), inhibited MAO A and B activities in the micro-molar concentrations (Patsenka and Antkiewicz-Michaluk 2004). The compounds had antioxidant properties as indicated by the ability of TIQ and 1MeTIQ to inhibit free radical formation and to abolish H_2_O_2_ generation from DA via the Fenton reaction (for review see Singer and Ramsay 1995; Antkiewicz-Michaluk et al. 2006). Those results demonstrate that TIQ and 1MeTIQ are MAO inhibitors and possess intrinsic antioxidant properties. In that light of these observations the question arises whether TIQ and 1MeTIQ may have antidepressant effect? Both compounds easily penetrate into the brain through the blood–brain barrier, and their neuroprotective properties might be relevant from the clinical point of view. Additionally, these compounds have not been investigated yet, in the context of their antidepressant properties in the reserpine model of depression.

In the present paper, we examined behavioral and neurochemical effects of acute and repeated treatment with a low dose of reserpine, and then we evaluated antidepressant-like effect of the investigated compounds, TIQ and 1MeTIQ, in reserpinized rats as a model of depression.

We used the behavioral forced swim test (FST) to examine the antidepressant properties of TIQ and 1MeTIQ. The FST is a test with high predictive validity for antidepressant efficacy in human depression. Recently, a behavior sampling technique was developed that scores individual response categories, including immobility, swimming, and climbing (Detke et al. 1995). Although all antidepressant drugs reduce immobility time in the FST, at least two distinct active behavioral patterns are produced by pharmacologically selective antidepressant drugs (Borsini and Meli 1988). Serotonin-selective re-uptake inhibitors increase swimming behavior, while drugs acting primarily to elevate extracellular levels of NA or DA increase climbing behavior (Borsini 1995; Detke et al. 1995; Detke and Lucki 1996). Additionally, the locomotor activity test was used to check motor function of reserpinized rats.

In the second part of the study, in addition to the behavioral tests, we carried out also the neurochemical *ex vivo* studies in the rat brain structures [ventral tegmental area (VTA), nucleus accumbens, and hypothalamus] to determine: the levels of monoamines and their metabolites, the rate of monoamine metabolism, and the indices of neuronal activity.

## Materials and Methods

### Animals

Behavioral tests were carried out on male Wistar rats (Charles River) of initial body weight 230–240 g (about 7 weeks old). The animals were kept under standard laboratory conditions with free access to laboratory food and tap water, at room temperature of 22 °C with an artificial day-night cycle (12/12 h, light on at 7 a.m.). All the procedures were carried out in accordance with the National Institutes of Health Guide for the Care and Use of Laboratory Animals and were granted an approval from the Bioethics Commission as compliant with Polish Law. The experimental protocols were approved by the Local Bioethics Commission of the Institute of Pharmacology, Polish Academy of Sciences in Kraków.

### Drugs

1,2,3,4-Tetrahydroisoquinoline hydrochloride (TIQ, Sigma-Aldrich, USA); 1MeTIQ were synthesized in Department of Drug Chemistry, Institute of Pharmacology Polish Academy of Sciences, Krakow, Poland), and the purity of the compound was verified by measurement of the melting point, and homogeneity was assessed on a chromatographic column. TIQ and 1MeTIQ were dissolved in sterile 0.9 % NaCl solution. The chemical structures of TIQ and 1MeTIQ are shown in Fig. 1. Reserpine (Sigma-Aldrich, USA) was suspended in 1 % Tween 80. The drugs were injected in a volume of 4 ml/kg.

### Treatments

In order to check pro-depressive effect of reserpine, the experimental protocol was divided into two main parts. In the first part of the study (reserpine model of depression), we analyzed reserpine-induced depressive disorder after a single and repeated administration (once daily for 14 days) of reserpine (0.2 mg/kg i.p.) in a variety of behavioral (forced swimming test, locomotor activity: travelled distance and rearing times), and biochemical tests (the concentration of monoamines: DA, NA, and serotonin in the brain structures). In order to evaluate the duration of the obtained reserpine effect, the tests were carried out 120 min after the last injection.

In the second part of that study, we evaluated the antidepressant-like effect of the investigated TIQs. For this purpose, TIQ and 1MeTIQ in a dose of 25 mg/kg i.p. were administered chronically (14 days) 30 min before each reserpine injection (0.2 mg/kg i.p.), and their effects on the reserpine-induced depressive-like disorder was investigated in the behavioral and biochemical tests 120 min after the last dose of reserpine. Control group received chronically 1 % Tween 80. Immediately after the end of behavioral tests, the rats were killed by decapitation, and different brain structures (VTA; nucleus accumbens and hypothalamus) were dissected for the later neurochemical studies that assessed the metabolism of monoamines by high pressure chromatography method (HPLC) with electrochemical detection (ED). The experiments were carried out between 10 a.m. and 4 p.m. Each experimental group consisted of 6-8 rats.

### Behavioral Studies

#### The FST Procedure

The studies were carried out on rats and were based on the method of Porsolt et al. (1978). All the animals were individually tested in the FST on two consecutive days with one session per day. On the first day, the rats were individually placed in non-transparent plastic cylinders (diameter: 23 cm, height: 50 cm) containing 30 cm of water, maintained at 25–26 °C. They were let to swim for 15 min before being removed (pre-test session). After that the animals were dried and returned to their home cages. The procedure was repeated 24 h later, and the time of the escape-oriented behavior of the rats was recorded (for 5-min test session). The observed behavioral parameters (in the order of priority) were: time spent floating in water (*immobility*), *swimming*, and struggling (*climbing)*. According to Detke et al. (1995), the immobility is described as behavior of the rat when it makes only the movements necessary to keep its head above the water. In this case, animals can make certain, slight swimming movements in order to remain afloat. Climbing is defined as vigorous movements of four limbs, with the front paws breaking against the wall of the cylinder. During swimming rats make coordinated and sustained movements (more than necessary) with all four limbs, usually traveling around the interior of the cylinder, but without breaking the surface of the water with forelimbs. Water was changed between subjects. The FST was performed 120 min after acute and chronic (14 days) administration of reserpine (0.2 mg/kg i.p.) In combined treatment groups, TIQ and 1MeTIQ (25 mg/kg i.p.) were administered chronically 30 min before each dose of reserpine.

#### Locomotor Activity

The locomotor activity was measured in actometers (Opto-Varimex activity monitors, Columbus Instruments, USA) linked on-line to an IBM-PC compatible computer. Each cage (43 x 44 x 25 cm) was surrounded with a 15 × 15 array of photocell beams located 3 cm from the floor surface. Interruptions of these photocell beams were counted as a measure of horizontal and vertical locomotor activity. Horizontal locomotor activity was defined as the travelled distance (in cm), and the vertical activity as rearing times (in seconds). Locomotor activity was analyzed using Auto-Track Software Program (Columbus Instruments, USA) and recorded in 15 min intervals for 60 min. Locomotor activity was measured at 120 min after acute and chronic administration (14 days) of reserpine (0.2 mg/kg i.p.).

### Neurochemical Studies

#### *Ex Vivo*: Monoamine Metabolism in Rat Brain Structures

The animals were killed by decapitation after the end of behavioral experiments. The brains were rapidly removed and dissected on an ice-cold glass plate. After decapitation, the VTA, nucleus accumbens, and hypothalamus were taken and immediately frozen on solid CO_2_ (−70 °C) until used for biochemical assays. DA and its metabolites, the intraneuronal, 3,4-dihydroxyphenylacetic acid (DOPAC); the extraneuronal, 3-methoxytyramine (3-MT), and the final metabolite, homovanillic acid (HVA); NA and its main extraneuronal brain metabolite, normetanephrine, (NM) and serotonin (5-HT) and its intraneuronal metabolite 5-hydroxyindolacetic acid (5-HIAA) were assayed by means of high-performance liquid chromatography (HPLC) with electrochemical detection (ED). The tissue samples were weighted and homogenized in ice-cold 0.1 M trichloroacetic acid containing 0.05 mM ascorbic acid. After centrifugation (10,000×*g*, 5 min), the supernatants were filtered through RC58 0.2 μm cellulose membranes (Bioanalytical Systems, West Lafayette, IN, USA). The chromatograph HP 1050 (Hewlett-Packard, Golden, CO, USA) was equipped with Hypersil columns BDS-C18 (4 × 100 mm, 3 μm). The mobile phase consisted of 0.05 M citrate–phosphate buffer, pH 3.5; 0.1 mM EDTA; 1 mM sodium octyl sulfonate; and 3.5 % methanol. The flow rate was maintained at 1 ml/min. DA, serotonin, NA, and their metabolites were quantified by peak area comparisons with standards run on the day of analysis (ChemStation, Hewlett-Packard software computer program).

### Calculations and Statistics

The data of behavioral and neurochemical studies were calculated by means of a one-way or two-way analysis of variance (ANOVA), followed when appropriate by Duncan’s post hoc test. The data were considered statistically significant when *P* < 0.05.

The total catabolism rate for DA was assessed from the ratio of the final DA metabolite concentration, HVA to DA concentration and expressed as the catabolic rate index [HVA]/[DA] × 100; the rate of DA MAO-dependent oxidation as the ratio: [DOPAC]/[DA] × 100; the rate of DA COMT-dependent *O*-methylation as the ratio: [3-MT]/[DA] × 100. Analogously, the rate of NA metabolism was expressed as the ratio of the extraneuronal metabolite normetanephrine to NA: [NM]/[NA] × 100 and serotonin as the ratio: [5-HIAA]/[5-HT] × 100. The indices were calculated using concentrations from individual tissue samples (Antkiewicz-Michaluk et al. 2001).

## Results

### Behavioral Studies

#### The Effect of Acute and Chronic Administration of Reserpine on the FST Carried out 120 min After the Last Injection

**Fig. 2:**
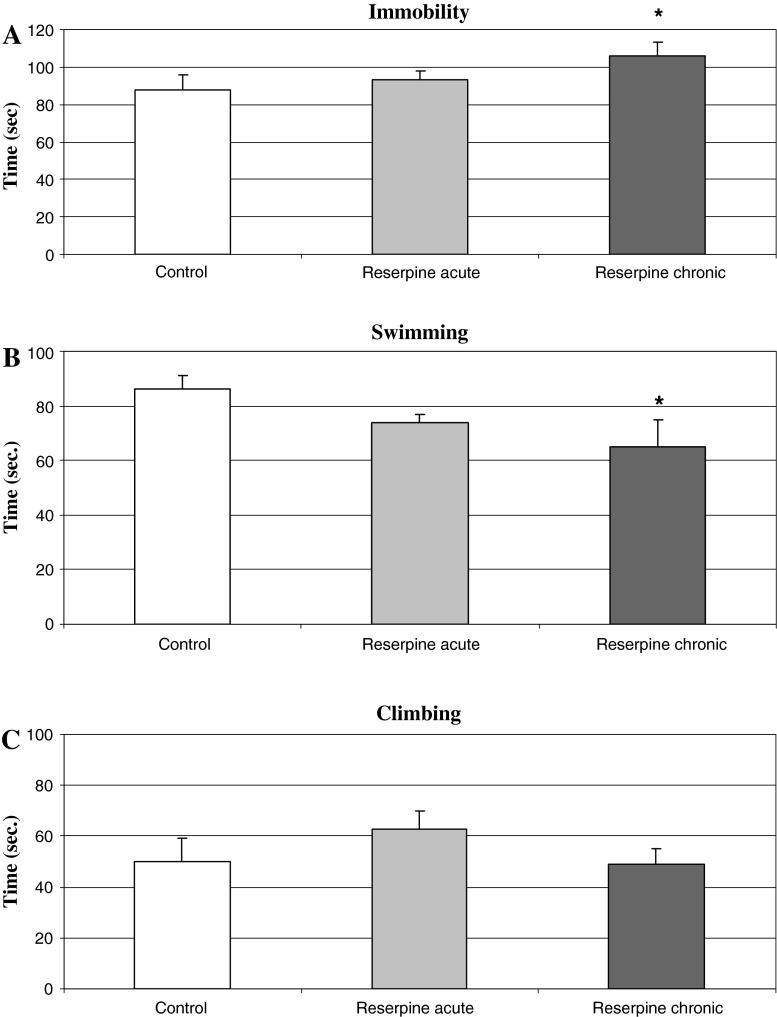
The effect of acute and repeated administration with a low dose of reserpine on FST in rat. Reserpine (0.2 mg/kg i.p.) was administered acute or chronically, once daily for 14 days. Control group received chronically 1 % Tween 80. FST was carried out 120 min after the last dose of reserpine. The data are the mean ± SEM. The results were analyzed by means of one-way ANOVA, followed when appropriate, by post hoc Duncan’s test. Statistical significance: *P < 0.05 versus control group

Chronic but not acute administration of reserpine (0.2 mg/kg i.p.) produced pro-depressive activity and significantly increased the immobility time in the FST in rats *F*_2,18_ = 2.66; *P* < 0.05 (Fig. 2a). The one-way ANOVA showed a significant decrease (~25 %) in the swimming activity after chronic reserpine *F*_2,18_ = 2.69; *P* < 0.05 and no change in the climbing (Fig. 2b, c).

#### The Locomotor Activity Test

**Fig. 3:**
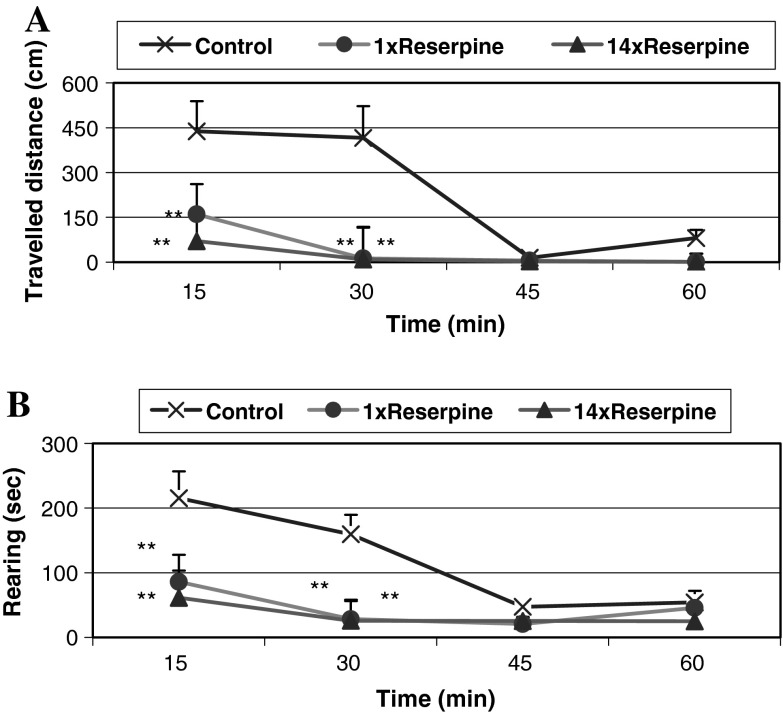
The effect of acute and repeated administration with a low dose of reserpine on the horizontal and vertical motor activity in rat. Reserpine (0.2 mg/kg i.p.) was administered acute or chronically, once daily for 14 days. Control group received chronically 1 % Tween 80. Horizontal locomotor activity was defined as the travelled distance (in cm) and the vertical activity as rearing times (in seconds). Locomotor activity was analyzed 120 min after acute and chronic treatment of reserpine (0.2 mg/kg i.p.) during 60 min using Auto-Track Software Program. The data are the mean ± SEM. The number of animals per group, *N* = 6–8. The results were analyzed by means of one-way ANOVA, followed when appropriate, by post hoc Duncan’s test. Statistical significance: **P < 0.001 versus control group

Both acute and chronic administration of reserpine in a low dose (0.2 mg/kg i.p.) produced a significant decrease in the horizontal (travelled distance in cm) and vertical (rearing time in seconds) exploratory locomotor activity of rats (*P* < 0.001) during the first 30 min after the start of the measurement of motor activity. At later intervals of 45 and 60 min, no changes in motor activity were detected between reserpine groups and the control (Fig. 3a, b).

#### The Effect of Chronic Administration TIQ and 1MeTIQ on Reserpine-Evoked Depressive-like Behavior in the FST in the Rat

**Fig. 4:**
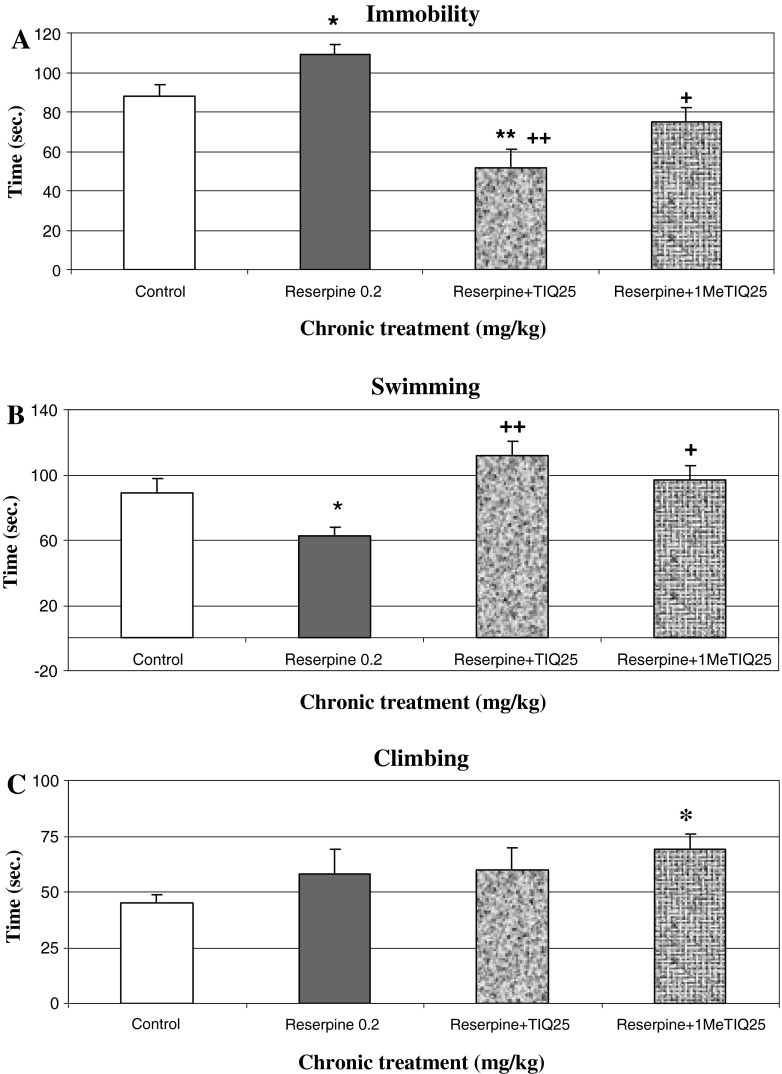
The effect of chronic administration TIQ and 1MeTIQ (25 mg/kg i.p.) on reserpine (0.2 mg/kg i.p.) evoked depressive-like effect in FST in rat. Reserpine (0.2 mg/kg i.p.) was administered chronically, once daily for 14 days. TIQ and 1MeTIQ (25 mg/kg i.p.) were administered 30 min before each dose of reserpine (combined groups). Control group received chronically 1 % Tween 80. FST was carried out 120 min after the last dose of reserpine. The data are the mean ± SEM. The results were analyzed by means of one-way ANOVA, followed when appropriate, by post hoc Duncan’s test. Statistical significance: *P < 0.05, **P < 0.01 versus control group. ^+^ *P* < 0.05, ^++^ *P* < 0.01 versus reserpine group

Chronic administration (14 days) of TIQ and 1MeTIQ in a dose of 25 mg/kg i.p. together with reserpine (0.2 mg/kg i.p.) produced antidepressant-like activity and completely antagonized pro-depressive effect of chronic reserpine (Fig. 4a, b). The one-way ANOVA showed a significant effect of treatments, *F*_3,21_ = 9.75; *P* < 0.0003, and Duncan’s post hoc test revealed an increase in the immobility time for reserpine alone versus control group (*P* < 0.05), and its significant decrease in both the combined groups: TIQ + reserpine (*P* < 0.01) and 1MeTIQ + reserpine (*P* < 0.05) (Fig. 4a). Similarly, a significant decrease in the swimming time in reserpine group was completely antagonized by TIQ and 1MeTIQ in the combined treatment groups, *F*_3,21_ = 4.47; *P* < 0.01 (Fig. 4b). The climbing time was significantly increased only in the combined treatment group 1MeTIQ + reserpine, *F*_3,21_ = 3.28; *P* < 0.05 (Fig. 4c).

### Neurochemical Studies

#### The Comparison of a Single and Repeated Administration of a Low Dose of Reserpine on the Concentration of Monoamines: DA, NA, and Serotonin in the Nucleus Accumbens and Hypothalamus

The one-way ANOVA showed a significant effect of chronic reserpine administration on DA *F*_2/15_ = 6.81, *P* < 0.002 and NA level *F*_2/15_ = 3.39, *P* < 0.03 in the nucleus accumbens. The Duncan’s post hoc test indicated that chronic treatment with reserpine in contrast to a single injection decreased significantly the level of DA (by ~35 % vs. control; *P* < 0.01) and NA (about 50 % of control; *P* < 0.05). Additionally, the one-way ANOVA demonstrated also a significant effect of chronic reserpine on DA *F*_2/15_ = 3.83, *P* < 0.02; NA *F*_2/15_ = 26.94, *P* < 0.0001; serotonin *F*_2/15_ = 16.85, *P* < 0.0007 concentrations in the hypothalamus. The Duncan’s post hoc test revealed a significant decrease in all monoamines: DA (about 40 % of control, *P* < 0.05); NA (30 % of control, *P* < 0.02) and serotonin (about 35 % of control, *P* < 0.01) after chronic reserpine and no effect of a single injection (Table 1).

**Table 1:** The comparison of a single and repeated administration of a low dose of reserpine on the concentration of monoamines: DA, NA, and serotonin in rat brain structures

Treatment (mg/kg)	*N*	DA (ng/g wt)	NA (ng/g wt)	Serotonin (ng/g wt)
Nucleus accumbens				
Control	6	9,601 ± 502	134 ± 11	419 ± 41
Reserpine acute	6	8,870 ± 388	102 ± 16	320 ± 27
Reserpine chronic	6	7,075 ± 445**^+^	76 ± 9*	359 ± 26
*F*		*F*_(2/15)_ = 6.81	*F*_(2/15)_ = 3.39	*F*_(2/15)_ = 1.83
		*P* < 0.002	*P* < 0.03	NS
Hypothalamus				
Control	6	511 ± 49	1,901 ± 70	796 ± 19
Reserpine acute	6	417 ± 28	1,806 ± 77	754 ± 27
Reserpine chronic	6	294 ± 77*	1,323 ± 46**^++^	509 ± 24**^++^
*F*		*F*_(2/15)_ = 3.73	*F*_(2/15)_ = 26.94	*F*_(2/15)_ = 16.85
		*P* < 0.02	*P* < 0.0000001	*P* < 0.000007

Reserpine (0.2 mg/kg i.p.) was administered once (acute treatment) and for 14 days once daily (chronic treatment). Control group received once daily for 14 days 1 % Tween 80. Animals were decapitated 120 min after the last injection of reserpine (acute and chronic treatment). The concentration (ng/g wet tissue) of monoamines: DA, NA, and serotonin was measured in the rat nucleus accumbens and hypothalamus. The data are the mean ± SEM. The results were analyzed by means of one-way ANOVA, followed when appropriate, by post hoc Duncan’s test

Statistical significance: * *P* < 0.05, ** *P* < 0.01 versus control group; ^+^ *P* < 0.05, ^++^ *P* < 0.01 versus reserpine acute

#### The Effect of Chronic Administration of TIQ and 1MeTIQ on Reserpine-Induced Changes in the DA System

##### Dopamine

The one-way ANOVA indicated a significant effect of treatment on DA concentration in all investigated structures: VTA (*F*_3/20_ = 3.68; *P* < 0.05), nucleus accumbens (*F*_3/20_ = 4.10; *P* < 0.02) and hypothalamus (*F*_3/20_ = 7.34; *P* < 0.001). The Duncan’s post hoc test revealed a significant decrease in DA concentration (from 20 to 40 % of control) in all investigated structures after chronic reserpine administration. The Duncan’s post hoc test demonstrated that TIQ and 1MeTIQ completely antagonized the reserpine-induced decrease in the DA concentration, and the level of DA returned to the control value in the combined treatment groups (Table 2).

**Table 2:** The effect of chronic administration of TIQ and 1MeTIQ on reserpine-induced changes in the level of DA and its metabolites in rat brain structures

Chronic treatment (mg/kg)	*N*	DA (ng/g wt)	DOPAC (ng/g wt)	3-MT (ng/g wt)	HVA (ng/g wt)
VTA					
Control	6	1,990 ± 269	594 ± 67	39 ± 8.7	362 ± 16
Reserpine 0.2	6	1,643 ± 70*	593 ± 36	40 ± 3.1	343 ± 22
Reserpine 0.2 + TIQ25	6	2,077 ± 139^**+**^	342 ± 24**^++^	79 ± 5.6*^+^	286 ± 15**^+^
Reserpine 0.2 + 1MeTIQ25	6	2,255 ± 150^**+**^	299 ± 42**^++^	94 ± 16.9**^++^	216 ± 17**^++^
*F*		*F*_(3/20)_ = 3.66	*F*_(3/20)_ = 12.30	*F*_(3/20)_ = 7.83	*F*_(3/20)_ = 13.39
		*P* < 0.05	*P* < 0.00008	*P* < 0.001	*P* < 0.00005
Nucleus accumbens					
Control	6	11,274 ± 433	1,979 ± 169	206 ± 19	862 ± 41
Reserpine 0.2	6	8,892 ± 254*	1,710 ± 53*	179 ± 22	558 ± 16**
Reserpine 0.2 + TIQ25	6	10,503 ± 645^**+**^	1,030 ± 73**^++^	196 ± 14	561 ± 63**
Reserpine 0.2 + 1MeTIQ25	6	11,521 ± 636^++^	1,202 ± 44**^++^	252 ± 39	581 ± 28**
*F*		*F*_(3/20)_ = 4.10	*F*_(3/20)_ = 18.77	*F*_(3/20)_ = 1.06	*F*_(3/20)_ = 13.00
		*P* < 0.02	*P* < 0.000005	NS	*P* < 0.00006
Hypothalamus					
Control	6	549 ± 31	97 ± 8.6	12 ± 0.7	45 ± 3.9
Reserpine 0.2	6	324 ± 17**	91 ± 10.4	10 ± 0.9	38 ± 3.2
Reserpine 0.2 + TIQ25	6	443 ± 36*^+^	50 ± 5.7**^++^	19 ± 3.5*^++^	39 ± 3.0
Reserpine 0.2 + 1MeTIQ25	6	419 ± 20*^+^	42 ± 1.0**^++^	18 ± 1.7^+^	25 ± 2.8**^++^
*F*		*F*_(3/20)_ = 7.34	*F*_(3/20)_ = 14.37	*F*_(3/20)_ = 4.41	*F*_(3/20)_ = 6.49
		*P* < 0.001	*P* < 0.00003	*P* < 0.01	*P* < 0.003

Reserpine (0.2 mg/kg i.p.) was administered chronically, once daily for 14 days. TIQ and 1MeTIQ (25 mg/kg i.p.) were administered 30 min before each dose of reserpine (combined groups). Control group received chronically 1 % Tween 80. Animals were decapitated 120 min after the last drug injection. The concentration of DA and its metabolites were expressed as ng/g wet tissue. The data are the mean ± SEM. The results were analyzed by means of one-way ANOVA, followed when appropriate, by post hoc Duncan’s test

Statistical significance: * *P* < 0.05, ** *P* < 0.01 versus control group. ^+^ *P* < 0.05, ^++^ *P* < 0.01 versus reserpine group

##### Dihydroxyphenylacetic Acid

The one-way ANOVA demonstrated a significant effect of treatment on DOPAC level in the VTA (*F*_3/20_ = 12.30; *P* < 0.00008), nucleus accumbens (*F*_3/20_ = 18.77; *P* < 0.00005), and hypothalamus (*F*_3/20_ = 14.37; *P* < 0.00003). The Duncan’s post hoc test indicated that TIQ and 1MeTIQ administered together with reserpine significantly (*P* < 0.01) decrease the DOPAC concentration versus control group in all investigated structures (Table 2).

##### 3-Methoxytyramine

The one-way ANOVA revealed a significant effect of treatment on 3-MT concentration in the VTA (*F*_3/20_ = 7.83; *P* < 0.001), and hypothalamus (*F*_3/20_ = 4.41; *P* < 0.01) but not in the nucleus accumbens (*F*_3/20_ = 1.06; NS). The Duncan’s post hoc test demonstrated that TIQ and 1MeTIQ administered together with reserpine produced the significant increase in 3-MT concentration versus control group in the VTA and hypothalamus (from 50 % up to 100 % of control, respectively) (Table 2).

##### Homovanillic Acid

The statistical analysis revealed a significant effect of treatment on the level of HVA in the VTA (*F*_3/20_ = 13.39; *P* < 0.00005), nucleus accumbens (*F*_3/20_ = 13.00; *P* < 0.00006), and hypothalamus (*F*_3/20_ = 7.83; *P* < 0.001). The Duncan’s post hoc test demonstrated that HVA level was significantly decreased (*P* < 0.01) by reserpine (only in the nucleus accumbens) and by TIQ and 1MeTIQ in the combined groups in the investigated structures (Table 2).

##### The Indices of DA Catabolism

The one-way ANOVA indicated a significant effect of treatment on the rate of total DA catabolism [HVA]/[DA] in the investigated structures: VTA (*F*_3/20_ = 8.19; *P* < 0.0009), nucleus accumbens (*F*_3/20_ = 17.51; *P* < 0.00008), and hypothalamus (*F*_3/20_ = 9.02; *P* < 0.0005) (Table 3). The Duncan’s post hoc test demonstrated the opposite effect of reserpine on the total DA catabolism: in the nucleus accumbens, it significantly decreased (about 25 % of control; *P* < 0.01), while in the hypothalamus, it significantly increased (about 50 % of control; *P* < 0.01), and there was no change in the VTA. An increase in the rate of total DA catabolism after reserpine in the hypothalamus was completely antagonized by TIQ and 1MeTIQ (Table 3).

**Table 3:** The effect of chronic administration of TIQ and 1MeTIQ on reserpine-induced changes in the rate of DA metabolism after chronic administration in rat brain structures

Chronic treatment (mg/kg)	*N*	[HVA]/[DA]	[DOPAC]/[DA]	[3-MT]/[DA]
VTA				
Control	6	20 ± 2.9	29 ± 1.2	2 ± 0.3
Reserpine 0.2	6	21 ± 0.9	36 ± 1.1**	2 ± 0.1
Reserpine 0.2 + TIQ25	6	14 ± 1.0*^+^	16 ± 0.8**^++^	4 ± 0.3**^++^
Reserpine 0.2 + 1MeTIQ25	6	10 ± 1.4**^++^	14 ± 1.7**^++^	4 ± 0.3**^++^
*F*		*F*_(3/20)_ = 8.19	*F*_(3/20)_ = 71.89	*F*_(3/20)_ = 14.54
		*P* < 0.0009	*P* < 0.0000001	*P* < 0.00002
Nucleus accumbens				
Control	6	8 ± 0.2	17 ± 1.4	2 ± 0.2
Reserpine 0.2	6	6 ± 0.2**	19 ± 0.5*	2 ± 0.2
Reserpine 0.2 + TIQ25	6	5 ± 0.4**^+^	10 ± 0.2**^++^	2 ± 0.1
Reserpine 0.2 + 1MeTIQ25	6	5 ± 0.2**^++^	10 ± 0.3**^++^	2 ± 0.3
*F*		*F*_(3/20)_ = 17.51	*F*_(3/20)_ = 34.88	*F*_(3/20)_ = 0.78
		*P* < 0.000008	*P* < 0.0000001	NS
Hypothalamus				
Control	6	8 ± 0.5	17 ± 0.7	2 ± 0.2
Reserpine 0.2	6	12 ± 0.8**	28 ± 1.8**	3 ± 0.2
Reserpine 0.2 + TIQ25	6	9 ± 0.8^+^	11 ± 0.5**^++^	5 ± 0.7**^+^
Reserpine 0.2 + 1MeTIQ25	6	7 ± 0.7^++^	12 ± 0.6**^++^	5 ± 0.3**^+^
*F*		*F*_(3/20)_ = 9.02	*F*_(3/20)_ = 53.42	*F*_(3/20)_ = 6.56
		*P* < 0.0005	*P* < 0.0000001	*P* < 0.002

Reserpine (0.2 mg/kg i.p.) was administered chronically, once daily for 14 days. TIQ and 1MeTIQ (25 mg/kg i.p.) were administered 30 min before each dose of reserpine (combined groups). Control group received chronically 1 % Tween 80. Animals were decapitated 120 min after chronic drugs administration. The rate of DA total metabolism was expressed as the ratio [HVA]/[DA] × 100; the rate of DA MAO-dependent oxidation as the ratio: [DOPAC]/[DA] × 100; and the rate of DA COMT-dependent O-methylation as the ratio: [3-MT]/[DA] × 100; and serotonin as the ratio: [5-HIAA]/[5-HT] × 100. The indices were calculated using concentrations from individual tissue samples. The data are the mean ± SEM. The results were analyzed by means of one-way ANOVA, followed when appropriate, by post hoc Duncan’s test

Statistical significance: * *P* < 0.05, ** *P* < 0.01 versus control group. ^+^ *P* < 0.05, ^++^ *P* < 0.01 versus reserpine-treated group

At the same time, the statistical analysis demonstrated a significant increase in the rate of DA oxidation, [DOPAC]/[DA] after chronic reserpine in all tested structures: VTA (*F*_3/20_ = 71.89; *P* < 0.00000), nucleus accumbens (*F*_3/20_ = 34.88; *P* < 0.00000) and hypothalamus (*F*_3/20_ = 53.42; *P* < 0.00000), total antagonism of this effect by TIQ, and 1MeTIQ in the combined groups (Table 3).

The rate of DA O-methylation, [3-MT]/[DA] was significantly elevated only by TIQ and 1MeTIQ in the combined treatment groups: in the VTA (*F*_3/20_ = 14.54; *P* < 0.0002) and in the hypothalamus (*F*_3/20_ = 6.56; *P* < 0.002).

#### The Effect of Chronic Administration of TIQ and 1MeTIQ on Reserpine-Induced Changes in the Noradrenergic System

##### Noradrenaline

The one-way ANOVA revealed a significant effect of treatment on NA concentration in the all investigated structures: VTA (*F*_3/20_ = 4.35; *P* < 0.01), nucleus accumbens (*F*_3/20_ = 3.39; *P* < 0.03), and hypothalamus (*F*_3/20_ = 3.62; *P* < 0.05). The Duncan’s post hoc test indicated a significant decrease in NA in the nucleus accumbens (about 45 % of control; *P* < 0.05) and in the hypothalamus (about 25 % of control; *P* < 0.05). TIQ and 1MeTIQ in the combined treatment groups completely antagonized the reserpine-induced depression in the concentration of NA (Table 4).

**Table 4:** The effect of chronic administration of TIQ and 1MeTIQ on reserpine-induced changes in the noradrenergic system after chronic administration in the different structures of rat brain

Chronic treatment (mg/kg)	*N*	NA (ng/g wt)	NM (ng/g wt)	[NM]/[NA]
VTA				
Control	6	606 ± 80	24 ± 4.3	4 ± 1.7
Reserpine 0.2	6	555 ± 48	16 ± 2.5*	3 ± 0.6
Reserpine 0.2 + TIQ25	6	801 ± 22*^+^	30 ± 3.6^++^	4 ± 0.4
Reserpine 0.2 + 1MeTIQ25	6	763 ± 62^++^	27 ± 2.0^+^	4 ± 0.4
*F*		*F*_(3/20)_ = 4.35	*F*_(3/20)_ = 3.61	*F*_(3/20)_ = 0.65
		*P* < 0.01	*P* < 0.03	NS
Nucleus accumbens				
Control	6	443 ± 48	15 ± 1.0	4 ± 0.8
Reserpine 0.2	6	233 ± 28*	9 ± 0.6*	4 ± 0.7
Reserpine 0.2 + TIQ25	6	575 ± 133^++^	16 ± 3.7^+^	3 ± 0.6
Reserpine 0.2 + 1MeTIQ25	6	477 ± 37^+^	13 ± 1.1	3 ± 0.3
*F*		*F*_(3/20)_ = 3.37	*F*_(3/20)_ = 2.13	*F*_(3/20)_ = 1.48
		*P* < 0.03	NS	NS
Hypothalamus				
Control	6	1,208 ± 57	16 ± 1.0	1 ± 0.1
Reserpine 0.2	6	926 ± 71*	10 ± 0.6*	1 ± 0.1
Reserpine 0.2 + TIQ25	6	1,133 ± 110^+^	67 ± 8.2**^++^	6 ± 0.9**^++^
Reserpine 0.2 + 1MeTIQ25	6	1,247 ± 95^+^	37 ± 3.0**^++^	3 ± 0.2**^++^
*F*		*F*_(3/20)_ = 3.61	*F*_(3/20)_ = 33.32	*F*_(3/20)_ = 40.13
		*P* < 0.05	*P* < 0.0000001	*P* < 0.0000001

Reserpine (0.2 mg/kg i.p.) was administered chronically, once daily for 14 days. TIQ and 1MeTIQ (25 mg/kg i.p.) were administered 30 min before each dose of reserpine (combined groups). Control group received chronically 1 % Tween 80. Animals were decapitated 120 min after chronic drugs administration. The concentration of NA and its metabolite, normetanephrine (NM) was expressed as ng/g wet tissue. The rate of NA metabolism was expressed as the ratio of the extraneuronal metabolite, normetanephrine to NA: [NM]/[NA] × 100. Analogously to DA, the indices were calculated using concentrations from individual tissue samples. The data are the mean ± SEM. The results were analyzed by means of one-way ANOVA, followed when appropriate, by post hoc Duncan’s test

Statistical significance: * *P* < 0.05, ** *P* < 0.01 versus control group. ^+^ *P* < 0.05, ^++^ *P* < 0.01 versus reserpine-treated group

##### Normetanephrine

The one-way ANOVA demonstrated a significant effect of treatment on NM level in the VTA (*F*_3/20_ = 3.65; *P* < 0.03) and hypothalamus (*F*_3/20_ = 33.32; *P* < 0.00000). The Duncan’s post hoc test indicated that reserpine led to a decrease in NM level in the brain structures, and TIQ and 1MeTIQ administered together with reserpine significantly antagonized this effect. Particularly in the hypothalamus, TIQ led to a strong increase (about 400 % of control group, *P* < 0.001) substantially exceeding the value of NM in the control group (Table 4).

##### The Index of NA Catabolism: [NM]/[NA]

The one-way ANOVA revealed a significant effect of treatment on the index of NA catabolism [NM]/[NA] only in the hypothalamus (*F*_3/20_ = 40.13; *P* < 0.00000). The Duncan’s post hoc test indicated that TIQ and 1MeTIQ in the combined groups strongly increased the index of NA catabolism (about 600 %, *P* < 0.001; and 300 %, *P* < 0.001; respectively) (Table 4).

#### The Effect of Chronic Administration of TIQ and 1MeTIQ on Reserpine-Induced Changes in the Serotonin System

##### Serotonin

The one-way ANOVA indicated a significant effect of treatment on the level of serotonin only in the hypothalamus (*F*_3/20_ = 6.95; *P* < 0.002). The Duncan’s post hoc test indicated that chronic administration of reserpine produced a significant decrease in serotonin concentration in the VTA (about 35 % of control, *P* < 0.05) and in the hypothalamus (about 30 % of control, *P* < 0.01). These effects were antagonized by TIQ and 1MeTIQ in the combined treatment groups (Table 5).

**Table 5:** The effect of chronic administration of TIQ and 1MeTIQ on reserpine-induced changes in the serotonin system after chronic administration in the different structures of rat brain

Chronic treatment (mg/kg)	*N*	5-HT (ng/g wt)	5-HIAA (ng/g wt)	[5-HIAA]/[5-HT]
VTA				
Control	6	430 ± 54	493 ± 61	116 ± 9.9
Reserpine 0.2	6	279 ± 19*	552 ± 37	200 ± 15.2**
Reserpine 0.2 + TIQ25	6	377 ± 41	345 ± 49*^++^	92 ± 8.5^++^
Reserpine 0.2 + 1MeTIQ25	6	344 ± 38	221 ± 19**^++^	67 ± 7.2**^++^
*F*		*F*_(3/20)_ = 2.48	*F*_(3/20)_ = 11.29	*F*_(3/20)_ = 29.39
		NS	*P* < 0.0001	*P* < 0.0000001
Nucleus accumbens				
Control	6	551 ± 44	439 ± 22	81 ± 4.0
Reserpine 0.2	6	558 ± 34	442 ± 24	79 ± 2.9
Reserpine 0.2 + TIQ25	6	574 ± 43	343 ± 14**^++^	62 ± 5.8**^++^
Reserpine 0.2 + 1MeTIQ25	6	616 ± 21	320 ± 17**^++^	52 ± 3.8**^++^
*F*		*F*_(3/20)_ = 0.63	*F*_(3/20)_ = 10.15	*F*_(3/20)_ = 10.63
		NS	*P* < 0.0002	*P* < 0.0002
Hypothalamus				
Control	6	746 ± 18	286 ± 7	38 ± 0.7
Reserpine 0.2	6	524 ± 32**	314 ± 15	61 ± 4.9**
Reserpine 0.2 + TIQ25	6	658 ± 54^+^	210 ± 29**^++^	32 ± 2.8^++^
Reserpine 0.2 + 1MeTIQ25	6	667 ± 26^+^	207 ± 9**^++^	31 ± 2.5^++^
*F*		*F*_(3/20)_ = 6.94	*F*_(3/20)_ = 9.54	*F*_(3/20)_ = 20.04
		*P* < 0.002	*P* < 0.0004	*P* < 0.000003

Reserpine (0.2 mg/kg i.p.) was administered chronically, once daily for 14 days. TIQ and 1MeTIQ (25 mg/kg i.p.) were administered 30 min before each dose of reserpine (combined groups). Control group received chronically 1 % Tween 80. Animals were decapitated 120 min after chronic drugs administration. The concentration of serotonin (5-HT) and its metabolite, 5-hydroxyindolacetic acid (5-HIAA) was expressed as ng/g wet tissue. The rate of serotonin metabolism was expressed as the ratio of its metabolite, 5-HIAA to serotonin: [5-HIAA]/[5-HT]x100. The indices were calculated using concentrations from individual tissue samples. The data are the mean ± SEM. The results were analyzed by means of one-way ANOVA, followed when appropriate, by post hoc Duncan’s test

Statistical significance: * *P* < 0.05, ** *P* < 0.01 versus control group. ^+^ *P* < 0.05, ^++^ *P* < 0.01 versus reserpine-treated group

##### 5-Hydroxyindolacetic acid

The one-way ANOVA demonstrated a significant effect of treatment on the level of 5-HIAA in the tested structures: VTA (*F*_3/20_ = 11.29; *P* < 0.00012), nucleus accumbens (*F*_3/20_ = 10.15; *P* < 0.0002), and hypothalamus (*F*_3/20_ = 9.54; *P* < 0.0004). The Duncan’s post hoc test indicated that repeated treatment of reserpine had no effect but chronic administration of TIQ and 1MeTIQ together with reserpine produced a significant (*P* < 0.01) decrease of 5-HIAA concentration in all tested structures (Table 5).

##### The Index of Serotonin Catabolism: [5-HIAA]/[5-HT]

The one-way ANOVA revealed a significant effect of treatment on the index of serotonin catabolism [5-HIAA]/[5-HT] in all structures: VTA (*F*_3/20_ = 29.39; *P* < 0.00000), nucleus accumbens (*F*_3/20_ = 10.65; *P* < 0.0002), and hypothalamus (*F*_3/20_ = 20.04; *P* < 0.00000). The Duncan’s post hoc test demonstrated that repeated treatment with reserpine significantly increased serotonin metabolic index [5-HIAA]/[5-HT] in the VTA (about 70 % of control, *P* < 0.01) and in the hypothalamus (about 60 % of control, *P* < 0.01) but did not change it in the nucleus accumbens. TIQ and 1MeTIQ significantly antagonized the effect evoked by reserpine in these structures and clearly decreased the rate of serotonin metabolism in the nucleus accumbens (from 25 to 30 % of control, respectively, *P* < 0.01) (Table 5).

## Discussion

In this study, we investigated the effects of repeated administration of a low dose of reserpine (0.2 mg/kg i.p.) on behavioral (FST, motor function) and neurochemical parameters, and then we studied the effect of TIQ and 1MeTIQ on reserpine-induced depression in the rat. In fact, we observed that chronic but not acute treatment with a low dose of reserpine induced a distinct depressive-like behavior in the FST, motor impairment, and additionally a significant decrease in the level of DA, NA, and serotonin in the brain (Figs. 2, 3; Table 1).

As already well known, reserpine is an inhibitor of VMAT2 and interferes with the storage of monoamines by blocking the ATP-dependent uptake mechanism of the storage organelles (Nagakura et al. 2009; Rojas-Corrales et al. 2004). In addition to that, the oxidative catabolism of cytosolic DA and serotonin by monoamine oxidase A and B (MAO) is accelerated, which is followed by disappearance of these neurotransmitters and formation of a cellular oxidant hydrogen peroxide (especially in DA MAO-dependent oxidation). This action mimics the increased turnover of DA in the surviving dopaminergic terminals in the course of PD (Gerlach and Riederer 1996). Interestingly, the VMAT2-deficient animals showed an increased oxidative stress, progressive loss of DA terminals, and accumulation of α-synuclein (Caudle et al. 2007, 2008). There are many studies showing that depression is characterized by a significantly decreased antioxidant status, as evidenced by a lowered tryptophan, tyrosine, vitamin E, zinc concentration, and reduced glutathione, which are all antioxidants (Maes et al. 2011; Kodydkova et al. 2009). Recently, a new hypothesis postulating that the activation of inflammatory and oxidative stress pathways is a key pathophysiological factor in depression, was formulated (Vetulani and Nalepa 2000; Maes 2008).

One of the main purposes of this study was to find out more realistic model of depression which could be correlated with neurochemical changes in monoaminergic systems for estimation antidepressant efficacy of the investigated new compounds: TIQ and 1MeTIQ. We applied a such small dose of reserpine (0.2 mg/kg) which because its low concentration only partially affected a vesicular monoamines T2 transporter, and after acute administration did not evoke any changes in the forced swim behavioral test as well as in the biochemical parameters (the concentration of monoamines) in the brain. Somehow, repeated (daily for 14 days) administration leads to the significant “depression–like” syndrome in FST with the simultaneous distinct drop of monoamine concentrations in the brain structures.

In the light of these observations, we can suggest that repeated treatment with a low dose of reserpine could be a good progressive model of depression and concomitant abnormalities in the motivation function and, additionally, a useful tool for testing potential new antidepressants. Our results are also in agreement with other findings showing that the reserpine model is characterized not only by behavioral depression but also by impairment of monoamine neurotransmission in the brain (Colpaert 1987; Gerlach and Riederer 1996; Fernandes et al. 2008, 2012). Previous in vitro studies have shown that the investigated compounds, TIQ and 1MeTIQ, possess free radical scavenging capacity and intrinsic antioxidant properties (Antkiewicz-Michaluk et al. 2006). Several TIQs and their congeners, including TIQ and 1MeTIQ, interfere with MAO activity, inducing putative neuroprotection related to the pathogenesis of PD (Naoi and Maruyama 1993). Both compounds investigated in the present study inhibited MAO A and MAO B activities with preferential effects on the MAO A form (Patsenka and Antkiewicz-Michaluk 2004). These results justify the question about the physiological significance of endogenous TIQs in the control of neurotransmitter function, and prevention of neurotoxicity related to MAO activity in the brain.

The FST measuring immobility has been shown to be an appropriate test to evaluate antidepressant activity, as antidepressants generally delay and decrease immobility (Cryan et al. 2005; Murray et al. 2008; Zhao et al. 2008). The modified FST measures the frequency of different types of active behaviors: swimming, which is sensitive to serotoninergic compounds, such as SSRIs, and climbing, which is sensitive to tricyclic antidepressants and drugs with selective effects on catecholamine transmission (Cryan and Lucki 2000; Cryan et al. 2005; Detke et al. 1995). As shown by Detke et al. (1995), the increase in climbing activity is connected with an enhanced NA system activation.

In the present study, we observed for the first time the antidepressant-like effect in the FST of the tetrahydroisoquinoline amines: TIQ and 1MeTIQ in the animal model of depressive disorder induced by repeated administration of reserpine. Chronic reserpine significantly increased the immobility time in the FST, and concomitantly produced a significant decrease in the swimming time. However, in the locomotor activity test, both acute and repeated administration of reserpine produced a significant depression in the horizontal (travelled distance in cm) and vertical (rearing time in sec.) exploratory locomotor activity of rats (*P* < 0.001) during the first 30 min after the start of the measurement of motor activity (Fig. 3a, b). On the contrary, in the FST, there was a clear distinction between the acute and chronic effects of reserpine administration, where only chronic treatment led to the depressive-like behavior in that test (Fig. 2). The results of these experiments clearly indicate a significant dissociation between “pro-depressive” effects observed in the FST (inhibition of motivation) only after repeated reserpine treatment and, in contrast to that, depression of the locomotor activity after both, acute and repeated administration. Taken together the results also indicate that locomotor activity test is not suitable for estimation “pro-depressive” effects in rat.

The investigated compounds, TIQ and 1MeTIQ, administered chronically together with a low dose of reserpine completely antagonized reserpine-produced depression as measured by both investigated parameters: immobility time and swimming time (Fig. 4). The behavioral data obtained in the FST clearly indicate that both compounds express antidepressant-like activity in reserpinized rat. The data are in agreement with our recent papers concerning antidepressant-like effect of TIQ and 1MeTIQ in the FST and chronic mild stress model, which was comparable to classic antidepressants, imipramine, and desipramine (Wąsik et al. 2013; Możdżeń et al. submitted to the Editor 2013). Neurochemical data showed that these effects may be connected with the activation of monoaminergic system (dopaminergic, serotoninergic, and noradrenergic) in the brain. As already well known, multiple mechanisms are responsible for the development of depression. Monoamine neurotransmitters are involved in the pathogenesis of depression and play an important role in mediating the effects of antidepressants (Javaid et al. 1979; Borsini and Meli 1988). It is well documented by clinical data that the classical antidepressants (imipramine, desipramine) as well as SSRI activate monoaminergic neurotransmission in the brain as reuptake inhibitors of NA and serotonin (Grunewald et al. 1979; Javaid et al. 1979; Borsini and Meli 1988; Borsini 1995; Cryan and Lucki 2000; Cryan et al. 2005; Detke et al. 1995).

Biochemical data demonstrated that chronic treatment with a low dose of reserpine in contrast to acute administration produced the depression of monoamines in the brain structures. The concentrations of DA (in all investigated structures), NA (in the nucleus accumbens and hypothalamus), and serotonin (in the VTA and hypothalamus) were significantly lowered (Tables 2, 4, 5). In opposite, the rate of MAO-dependent oxidation of DA and serotonin was significantly increased (Tables 3, 5). These biochemical effects obtained after repeated reserpine administration were completely antagonized by chronic joint injections of TIQ or 1MeTIQ with reserpine. Regarding the mechanism of action of TIQs in reversing the effects of reserpine, it was shown previously in our *ex vivo* experiments that both compounds, TIQ and 1MeTIQ, shifted DA catabolism from MAO-dependent oxidation to COMT-dependent methylation and abolished at least generation of hydroxyl radicals via Fenton reaction (Antkiewicz-Michaluk et al. 2006). Such MAO-dependent oxidation inhibiting effect produced by TIQ and 1MeTIQ in reserpine-treated rats is clearly visible both in the decline of DA and serotonin metabolites (DOPAC and 5-HIAA, respectively) as well as in the decrease in oxidation indices in the combined treatment groups (Tables 2, 3, 5).

Similarly to DA and serotonin, both TIQ compounds normalized the level of NA and its extraneuronal metabolite, NM decreased by chronic administration of reserpine. NA released into the synaptic cleft is catabolized by COMT to NM by the process of COMT-dependent methylation, so this extraneuronal metabolite is a good marker of NA release. As it was demonstrated in the present paper, chronic reserpine inhibited noradrenergic transmission. TIQ and 1MeTIQ administered together with reserpine prevented the noradrenergic depression evoked by reserpine (Table 4).

Our present data demonstrate that the investigated compounds, TIQ and 1MeTIQ, used in the reserpine model of depression in the rat elicited the antidepressant-like activity in the FST. Both compounds are characterized by a wide spectrum of actions on all monoaminergic systems in the rat brain. Thanks to their ability to inhibit both MAO A and MAO B activity (Patsenka and Antkiewicz-Michaluk 2004) and to scavenge free radicals (Antkiewicz-Michaluk et al. 2006), TIQ and its close methyl derivative, 1MeTIQ may be useful not only for the therapy of neurodegenerative disease (e.g., PD) but also in the treatment of the depression as new antidepressants. Especially, 1MeTIQ raises hope for its application in depression as a safe drug with proven clinically useful mechanism of action described recently as neuroprotectant with antiaddictive potency (Antkiewicz-Michaluk et al. 2014).

## References

[CR1] Antkiewicz-Michaluk L, Michaluk J, Mokrosz M, Romańska I, Lorenc-Koci E, Otha S, Vetulani J (2001). Different action on dopamine catabolic pathways of two endogenous 1,2,3,4-tetrahydroisoquinolines with similar antidopaminergic properties. J Neurochem.

[CR2] Antkiewicz-Michaluk L, Lazarewicz JW, Patsenka A, Kajta M, Zieminska E, Salinska E, Wasik A, Golembiowska K, Vetulani J (2006). The mechanism of 1,2,3,4-tetrahydroisoquinolines neuroprotection: the importance of free radicals scavenging properties and inhibition of glutamate-induced excitotoxicity. J Neurochem.

[CR3] Antkiewicz-Michaluk L, Wąsik A, Michaluk J (2014) 1-Methyl-1,2,3,4-tetrahydroisoquinoline, an endogenous amine with unexpected mechanism of action: new vistas of therapeutic application. Neurotox Res 25:1–12 10.1007/s12640-013-9402-7 PMC3889699 23719903

[CR4] Borsini F (1995). Role of the serotonergic system in the forced swimming test. Neurosci Biobehav Rev.

[CR5] Borsini F, Meli A (1988). Is the forced swimming test a suitable model for revealing antidepressant activity?. Psychopharmacology.

[CR6] Cantello R, Aguggia M, Gilli M, Delsedime M, Cutin IC, Riccio A, Mutani R (1989). Major depression in Parkinson’s disease and the mood response to intravenous methylphenidate: possible role of the “hedonic” dopamine synapse. J Neurol Neurosurg Psychiatry.

[CR7] Caudle WM, Richardson JR, Wang MZ, Taylor TN, Guillot TS, McCormak AL (2007). Reduced vesicular storage of dopamine causes progressive nigrostriatal neurodegeneration. J Neurosci.

[CR8] Caudle WM, Colebrooke RE, Emson PC, Miller GW (2008). Altered vesicular dopamine storage in Parkinson’s disease: a premature demise. Trends Neurosci.

[CR9] Chan-Palay V, Asan E (1989). Alterations in catecholamine neurons of the locus coeruleus in senile dementia of Alzheimer type and in Parkinson’s disease with and without dementia and depression. J Comp Neurol.

[CR10] Colpaert FC (1987). Pharmacological characteristics of tremor, rigidity and hypokinesia induced by reserpine in rats. Neuropharmacology.

[CR11] Cryan JF, Lucki I (2000). Antidepressant-like behavioral effects mediated by 5-hydroxytryptamine(2C) receptors. J Pharmacol Exp Ther.

[CR12] Cryan JF, Page ME, Lucki I (2005). Differential behavioral effects of the antidepressants reboxetine, fluoxetine, and moclobemide in a modified forced swim test following chronic treatment. Psychopharmacol.

[CR13] Detke MJ, Lucki I (1996). Detection of serotonergic and noradrenergic antidepressants in the rat forced swimming test: the effects of water depth. Behav Brain Res.

[CR14] Detke MJ, Rickels M, Lucki I (1995). Active behaviors in the rat forced swimming test differentially produced by serotoninergic and noradrenergic antidepressants. Psychopharmacol (Berl)..

[CR15] Elhwuegi AS (2004). Central monoamines and their role in major depression. Prog Neuropsychopharmacol Biol Psychiatry.

[CR16] Fernandes VS, Ribeiro AM, Melo TG, Godinho M, Barbosa FF, Medeiros DS (2008). Memory impairment induced by low doses of reserpine in rats: possible relationship with emotional processing deficits in Parkinson’s disease. Prog Neuropsychopharmacol Biol Psychiatry.

[CR17] Fernandes VS, Santos JR, Leao A, Medeiros A, Melo TG, Izidio GS, Cabral A, Ribeiro RA (2012). Repeated treatment with a low dose of reserpine as a progressive model of Parkinson’s disease. Behav Brain Res.

[CR18] Gerlach M, Riederer P (1996). Animal models of Parkinson’s disease: an empirical comparison with the phenomenology of the disease in man. J Neural Transm.

[CR19] Graham DG (1978). Oxidative pathways for catecholamines in the genesis of neuromelanin and cytotoxic quinones. Mol Pharmacol.

[CR20] Grunewald GL, Reitz TJ, Ruth JA, Vollmer S, Eiden LE, Ruthedge CO (1979). Inhibition of neuronal uptake of ^3^H-biogenic amines rat cerebral cortex by partially and fully saturated derivatives of imipramine and desipramine. The importance of the aromatic ring in adrenergic amines—Part 3. Biochem Pharmacol.

[CR21] Javaid JI, Perel JM, Davis JM (1979). Inhibition of biogenic amines uptake by imipramine, desipramine, 2 OH-imipramine and 2 OH-desipramine in rat brain. Life Sci.

[CR22] Kandel ER, Kandel ER (2000). Disorders of mood: depression, mania, and anxiety disorders. Principles of neural science.

[CR23] Kodydkova J, Vavrova L, Zeman M, Jirak R, Macasek J, Stankova B, Tvrzicka E, Zak A (2009). Antioxidative enzymes and increased oxidative stress in depressive women. Clin Biochem.

[CR24] Kotake Y, Tasaki Y, Makino Y, Otha S, Hirobe M (1995). 1-Benzyl-1,2,3,4-tetrahydroisoquinoline as a parkinsonism-inducing agent: a novel endogenous amine in mouse brain and parkinsonian CSF. J Neurochem.

[CR25] Liu Y, Peter D, Roghani A (1992). cDNA that suppresses MPP toxicity encodes a vesicular amine transporter. Cell.

[CR26] Maes M (2008). The cytokine hypothesis of depression: inflammation, oxidative and nitrosative stress and leaky gut as a new targets for adjunctive treatments in depression. Neuro Endocrinol Lett.

[CR27] Maes M, Galecki P, Chang YS, Berk M (2011). A review on the oxidative and nitrosative stress pathways in major depression and their possible contribution to the (neuro)degenerative processes in that illness. Progress in NeuroPsychopharmacol Biol Psychiatry.

[CR28] Makino Y, Tasaki Y, Ohta S, Hirobe M (1990). Confirmation of the enantiomers of 1-methyl-1,2,3,4-tetrahydroisoquinoline in the mouse brain and foods applying gas chromatography/mass spectrometry with negative ion chemical ionization. Biomed Environ Mass Spectrom.

[CR29] Mayeux R, Stern Y, Cote L, Williams JB (1984). Altered serotonin metabolism in depressed patients with Parkinson’s disease. Neurology.

[CR30] McNaught KS, Carrupt PA, Altomare C, Cellamare S (1998). Isoquinoline derivatives as endogenous neurotoxins in the aetiology of Parkinson’s disease. Biochem Pharmacol.

[CR31] Miller GW, Gainetdinov RR, Levey AI, Caron MG (1999). Dopamine transporters and neuronal injury. Trends Pharmacol Sci.

[CR32] Murray F, Smith DW, Huston PH (2008). Chronic low dose corticosterone exposure decreased hippocampal cell proliferation, volume and induced anxiety and depression like behaviors in mice. Eur J Pharmacol.

[CR33] Nagakura Y, Oe T, Aoki T, Matsuoka N (2009). Biogenic amine depletion causes chronic muscular pain and tactile allodynia accompanied by depression: a putative animal model of fibromyalgia. Pain.

[CR34] Naoi M, Maruyama W (1993). Type B monoamine oxidase and neurotoxins. Eur Neurol.

[CR35] Patsenka A, Antkiewicz-Michaluk L (2004). Inhibition of rodent brain monoamine oxidase and tyrosine hydroxylase by endogenous compounds—1,2,3,4-tetrahydroisoquinoline alkaloids. Pol J Pharmacol.

[CR36] Porsolt RD, Anton G, Blavet N, Jalfre M (1978). Behavioural despair test in rats: a new model sensitive to antidepressant treatments. Eur J Pharmacol.

[CR37] Rojas-Corrales MO, Berrocoso E, Gibert-Rahola J, Mico JA (2004). Antidepressant-like effect of tramadol and its enantiomers in reserpinized mice: comparative study with desipramine, fluvoxamine, venlafaxine and opiates. J Psychopharmacol.

[CR38] Rommelspacher H, Susilo R (1985). Tetrahydroisoquinolines and β-carbolines: putative natural substances in plants and mammals. Prog Drug Res.

[CR39] Singer TP, Ramsay RR (1995). Flavoprotein structure and mechanism 2. Monoamine oxidases: old friends hold many surprises. FASEB J.

[CR40] Surratt CK, Persico AM, Yang DX (1993). A human synaptic vesicle monoamine transporter cDNA predicts posttranslational modifications, reveals chromosome 10 gene localization and identifies TaqI RFLPs. FEBS Lett.

[CR41] Tasaki Y, Makino Y, Ohta S, Hirobe M (1991). 1-Methyl-1,2,3,4-tetrahydroisoquinoline, decreasing in 1-methyl-4-phenyl-1,2,3,6-tetrahydropyridine-treated mouse, prevents parkinsonism-like behavior abnormalities. J Neurochem.

[CR42] Taylor TN, Caudle WM, Shepherd KR (2009). Nonmotor symptoms of Parkinson’s disease revealed in an animal model with reduced monoamine storage capacity. J Neurosci.

[CR43] Uhl GR (1998). Hypothesis: the role of dopaminergic transporters in selective vulnerability of cells in Parkinson’s disease. Ann Neurol.

[CR44] Vetulani J, Nalepa I (2000). Antidepressants: past, present and future. Eur J Pharmacol.

[CR45] Wąsik A, Romańska I, Antkiewicz-Michaluk L (2009). 1-Benzyl-1,2,3,4-tetrahydroisoquinoline, an endogenous parkinsonism-inducing toxin, strongly potentiates MAO-dependent dopamine oxidation and impairs dopamine release: ex vivo and in vivo neurochemical studies. Neurotox Res.

[CR46] Wąsik A, Możdżeń E, Romańska I, Michaluk J, Antkiewicz-Michaluk L (2013). Antidepressant-like activity of an endogenous amine, 1-methyl-1,2,3,4-tetrahydroisoquinoline in the behavioral despair test in the rat, and its neurochemical correlates: a comparison with the classical antidepressant, imipramine. Eur J Pharmacol.

[CR48] Zhao Y, Ma R, Shen J, Su H, Xing D, Du L (2008). A mouse model of depression induced by repeated corticosterone injections. Eur J Pharmacol.

[CR49] Ziemssen T, Reichmann H (2007). Non-motor dysfunction in Parkinson’s disease. Parkinsonism Relat Disord.

